# Critical Assessment of Large Language Models’ (ChatGPT) Performance in Data Extraction for Systematic Reviews: Exploratory Study

**DOI:** 10.2196/68097

**Published:** 2025-09-11

**Authors:** Hesam Mahmoudi, Doris Chang, Hannah Lee, Navid Ghaffarzadegan, Mohammad S Jalali

**Affiliations:** 1 MGH Institute for Technology Assessment, Harvard Medical School, 125 Nashua St, Boston, MA, 02114, United States, 1 6177243738; 2 Industrial and System Engineering Department, Virginia Tech, Falls Church, VA, United States

**Keywords:** large language models, generative artificial intelligence, systematic reviews, evidence synthesis, human-AI collaboration

## Abstract

**Background:**

Systematic literature reviews (SLRs) are foundational for synthesizing evidence across diverse fields and are especially important in guiding research and practice in health and biomedical sciences. However, they are labor intensive due to manual data extraction from multiple studies. As large language models (LLMs) gain attention for their potential to automate research tasks and extract basic information, understanding their ability to accurately extract explicit data from academic papers is critical for advancing SLRs.

**Objective:**

Our study aimed to explore the capability of LLMs to extract both explicitly outlined study characteristics and deeper, more contextual information requiring nuanced evaluations, using ChatGPT (GPT-4).

**Methods:**

We screened the full text of a sample of COVID-19 modeling studies and analyzed three basic measures of study settings (ie, analysis location, modeling approach, and analyzed interventions) and three complex measures of behavioral components in models (ie, mobility, risk perception, and compliance). To extract data on these measures, two researchers independently extracted 60 data elements using manual coding and compared them with the responses from ChatGPT to 420 queries spanning 7 iterations.

**Results:**

ChatGPT’s accuracy improved as prompts were refined, showing improvements of 33% and 23% between the initial and final iterations for extracting study settings and behavioral components, respectively. In the initial prompts, 26 (43.3%) of 60 ChatGPT responses were correct. However, in the final iteration, ChatGPT extracted 43 (71.7%) of the 60 data elements, showing better performance in extracting explicitly stated study settings (28/30, 93.3%) than in extracting subjective behavioral components (15/30, 50%). Nonetheless, the varying accuracy across measures highlighted its limitations.

**Conclusions:**

Our findings underscore LLMs’ utility in extracting basic as well as explicit data in SLRs by using effective prompts. However, the results reveal significant limitations in handling nuanced, subjective criteria, emphasizing the necessity for human oversight.

## Introduction

Systematic literature reviews (SLRs) are indispensable across various fields, synthesizing evidence to inform decision-making in areas as diverse as public health, policy, and biomedical sciences, where rigor and comprehensiveness are paramount. With the rapid expansion of the literature, SLRs are more important than ever to help not only synthesize evidence but also identify areas in which the literature is robust or deficient [1]. However, conducting SLRs is resource intensive, involving manual and careful screening of potentially relevant studies [2]. In particular, SLRs that assess and report on analytical methods and key findings require more domain-specific expertise and multiple researchers for coding, making them more challenging.

Given the recent rapid advancement of large language models (LLMs), researchers have proposed their potential utility in conducting SLRs [3,4]. Several reviews have found that artificial intelligence (AI)–enabled methods exhibit reasonable performance and improved efficiency in literature screening [5-9], an integral component of SLRs. In particular, the role of SLRs in health and biomedical sciences underscores the need for reliable, accurate data extraction tools that maintain the rigorous standards expected in these fields. However, studies that have tested the capabilities of AI for data extraction have identified challenges necessitating human intervention for completion [6,8].

To understand whether recently developed LLMs can overcome this barrier, many studies have evaluated the performance of various LLMs in automating SLR tasks [10-12]. Their findings reveal LLMs’ potential in extracting data that are relatively easily retrievable (ie, study design, participant characteristics, and primary outcomes). However, these studies have not explored the ability of LLMs to extract more complex data (eg, methods used to obtain study outcomes), which may pose greater challenges as extracting such complex information is often subject to individual researchers’ perspectives [13,14].

LLMs currently demonstrate advantages in assisting researchers, such as having rapid response times and providing high-level summaries of results [15]; however, a recent study revealed the shortcomings in ChatGPT’s depth of knowledge and contextual understanding for conducting SLRs in comparison to researchers [16]. Yet, there is growing potential for ChatGPT’s utility as an assistant in complex qualitative content analysis, as shown by a recent study that assessed its ability to categorize strategies and behaviors in forum posts about reducing sugar intake [17]. Thus, although LLMs show potential in data extraction tasks for SLRs, there is more to be explored on the current LLMs’ capability to undertake a comprehensive approach that involves extracting not only basic study characteristics but also information critical for interpreting the results of studies within SLRs.

In this study, we aimed to evaluate an LLM’s (ChatGPT) ability to extract more complex, nuanced data from scientific studies, representing a novel approach that goes beyond the simpler tasks of extracting descriptive information, such as study design or participant characteristics, which have been the primary focus of prior research. Our study applied a structured series of prompts and validations to systematically gauge where LLMs excel and where human oversight remains essential.

We used ChatGPT (GPT-4) for its accessibility and ease of use, recognizing that most individuals conducting SLRs may not be proficient with more customizable, technical platforms, such as the GPT application programming interface (API). Sophisticated LLM frameworks (eg, retrieval-augmented generation) offer technical capabilities that may better address needs for SLRs, but these features generally require AI-specific expertise. Consequently, our study prioritized a more common usage scenario in which a widely accessible GPT model is used for data extraction tasks.

## Methods

### Overview

We focused on COVID-19 simulation modeling studies as a case study, leveraging a large collection of study reports that our team recently assembled for a SLR that aimed to assess the incorporation of human behavior dynamics in COVID-19 simulation models [18]. For this study, the data elements selected for extraction were aligned with the specific objectives of our review: to evaluate ChatGPT’s capacity to manage both explicit and nuanced data in SLRs. To determine whether LLMs can effectively screen papers and extract information, we randomly chose 10 of the papers and extracted data both manually and using ChatGPT. We selected a sample of 10 studies [19-28] as this number was manageable for an in-depth exploration of each paper, allowing us to conduct detailed comparisons of extraction accuracy and making it feasible to perform multiple iterations of prompt engineering and assessment throughout the process.

Our examination of COVID-19 modeling studies leveraged our team’s expertise and enabled us to confidently determine correct answers for meaningful comparisons with ChatGPT’s outputs. Although COVID-19 may no longer be at the forefront of global attention, these studies remain a *methodologically rich test case* rather than a focus of current clinical relevance. They combine straightforward features (eg, study design, interventions) with more abstract elements of human behavior (eg, compliance with public health measures, mobility changes), allowing us to systematically assess ChatGPT’s performance across varying levels of complexity. Furthermore, the pandemic’s urgency previously highlighted the value of accurate, rapid synthesis of research findings during health emergencies, making it an ideal context for evaluating the practical utility of LLMs in accelerating SLRs.

Many researchers who conduct SLRs may not necessarily have the expertise required to implement LLMs through advanced tools. Thus, we conducted our analysis using ChatGPT, given that it is one of the most widely adopted models, attracting 393 million users each month, as shown by October 2024 data [29]. We used the web browser interface for its user-friendly design, as opposed to an API. Although using the API might have offered more controllable responses, our study prioritized accessibility and usability, reflecting the real-world context in which many researchers engage with ChatGPT. We selected the GPT-4 model specifically, as at the time of our analysis, it was one of the few that directly analyzed full text as a PDF file. Although our approach could also be applied to studies available in other formats, our focus on PDF files reflects their frequent use in academic publishing and SLRs.

### Ethical Considerations

This study did not involve human participants, identifiable human data, or interaction with individuals. As such, it did not fall under the scope of research requiring review by an institutional review board, and ethical approval was not required.

### Data Elements

We defined the sets of measures to be extracted as (1) study settings (ie, analysis location, modeling approach, and analyzed interventions) and (2) behavioral components (ie, changes in travel and mobility, perception of risk and severity, and compliance and resistance to public health measures). Therefore, for each of the 10 studies [19-28], we extracted 6 distinct data elements, resulting in 60 data elements.

We distinguished these measures to reflect their nature: study settings are straightforward, whereas extracting information on behavioral components in COVID-19 models is influenced by researchers’ perspectives [13]. We confined our study settings to information explicitly stated in the text. For behavioral components, we categorized them into no mention (A), mentioned but not modeled (B), modeled exogenously but not analyzed (C), modeled exogenously and analyzed (D), modeled endogenously but not analyzed (E), and modeled endogenously and analyzed (F). Endogenous modeling incorporated human behavior as an internal part of the model, where it both influenced and was influenced by the spread of COVID-19. Exogenous modeling indicated behavioral changes were external factors impacting the spread of COVID-19 without being influenced by it. This classification ranged from minimal (A) to comprehensive (F) incorporation into COVID-19 models.

### Data Extraction Process and Comparison

Two trained researchers independently extracted data from the 10 studies [19-28] and then reconciled discrepancies in their findings—6 related to study settings and 11 to behavioral components—for convergence. The researchers discussed any unresolved discrepancies with a third senior researcher to reach a consensus. Subsequently, we initiated a dedicated session for every study and prompt, uploading the individual files (in PDF) into the GPT-4 model using ChatGPT’s user interface (accessed in January-April 2024) and documented the responses. To gain confidence in our manual screening, wherever ChatGPT, consistently through iterations of prompts, provided answers that disagreed with our manual coding, we reassessed our original codings. Following this reassessment and after making necessary adjustments, we finalized the manual screening results and considered them as the correct responses. We calculated individual researchers’ average accuracy rates (the percentage of their correct responses before any consensus was reached), allowing us to directly compare ChatGPT’s performance against the individual researchers’ average accuracy for each measure throughout prompt iterations [8].

### Prompt Engineering

We started by providing ChatGPT with a general prompt to extract each desired data element. Due to initially unsatisfactory results, we iteratively engineered prompts based on the initial responses and our manual coding. This process involved altering the wording of prompts for clarity and concisely adding specific descriptions of our objective, approach, and definitions of key terms [30]. Particularly, we often guided ChatGPT to base any interpretations strictly on what was explicitly stated in the text, as it often made incorrect inferences. Furthermore, we followed up with ChatGPT regarding its incorrect responses by inquiring about potential improvements to the prompts after clarifying the desired answers [30].

We continued to refine the prompts until we achieved complete alignment with our manual screening results, reached saturation in improvement, or explored viable avenues for prompt enhancement to the best of our capabilities. Given the exploratory nature of this study and the fact that LLMs are designed to interact with users in real time, prompt refinement without a formal training phase reflects a common use case for this technology. Recent tutorials and case studies have demonstrated ChatGPT’s feasibility in domain-specific and rapid literature reviews, reinforcing its relevance as a practical, real-world tool that benefits from iterative, user-guided prompt refinement [31,32]. Table 1 illustrates an example of the iterative process used to prompt ChatGPT for extracting data elements in this study. We applied a similar method to extract additional data elements, with the processes reported in Multimedia Appendix 1.

**Table 1.** Example of the iterative process of prompt engineering to extract data elements.

Version	Description of prompt modification	Prompt
1	Initial prompt	What is the simulation modeling approach used in this paper?
2	Improved clarity	What model is used?
3	Focused output	Specify the overall type of model used in the study. If the modeling approach is unspecified, please state so.
4	Avoid overreporting	Specify the overall type (as opposed to the name) of the model used in the study. If the paper does not explicitly introduce the type of model used, state so by returning “unspecified.”
5	Exclude inferred information	Specify the overall type (as opposed to the name) of the model used in the study. If the PDF file does not explicitly introduce the type of model used, state so by returning “unspecified,” and do not infer the type of model.
6	Emphasis on explicit information	Read this PDF file line by line. Only specify the overall general type, as opposed to the name, of the model used in the study. If the author(s) of this PDF file do not explicitly introduce the type of model used, state so by returning “unspecified,” and do not infer the type of model. Be sure to only specify the type of foundational analytical model rather than any supplementary methods.
7	Step-by-step instructions	Read the provided PDF document line by line, focusing on identifying the general category or type of model mentioned in the study. Your task is to: Identify the type of model: Look for any mention of the foundational analytical model used in the research. Specify only the general category or type (eg, regression model) rather than the specific name or variant. Explicit mention required: If the document does not explicitly mention the type of model used in the analysis, respond with “unspecified.” Avoid making inferences based on the context or the data presented. Focus on the foundational model: Concentrate on identifying the primary analytical model that the study is based on. Disregard any supplementary methods, tools, or analytical techniques that are mentioned unless they are integral to the foundational model itself.

## Results

Table 2 summarizes ChatGPT’s responses across 6 measures for the last iteration of prompts. These measures were divided into study settings and behavioral components.

Through the course of iterations, we identified 4 instances where ChatGPT consistently disagreed with our manual coding but was determined to have provided the correct answer upon reassessment. These instances were among the prompts related exclusively to study settings: 3 pertained to answers generated for prompts about interventions analyzed, and 1 addressed the correct location of analysis.

**Figure 1.**
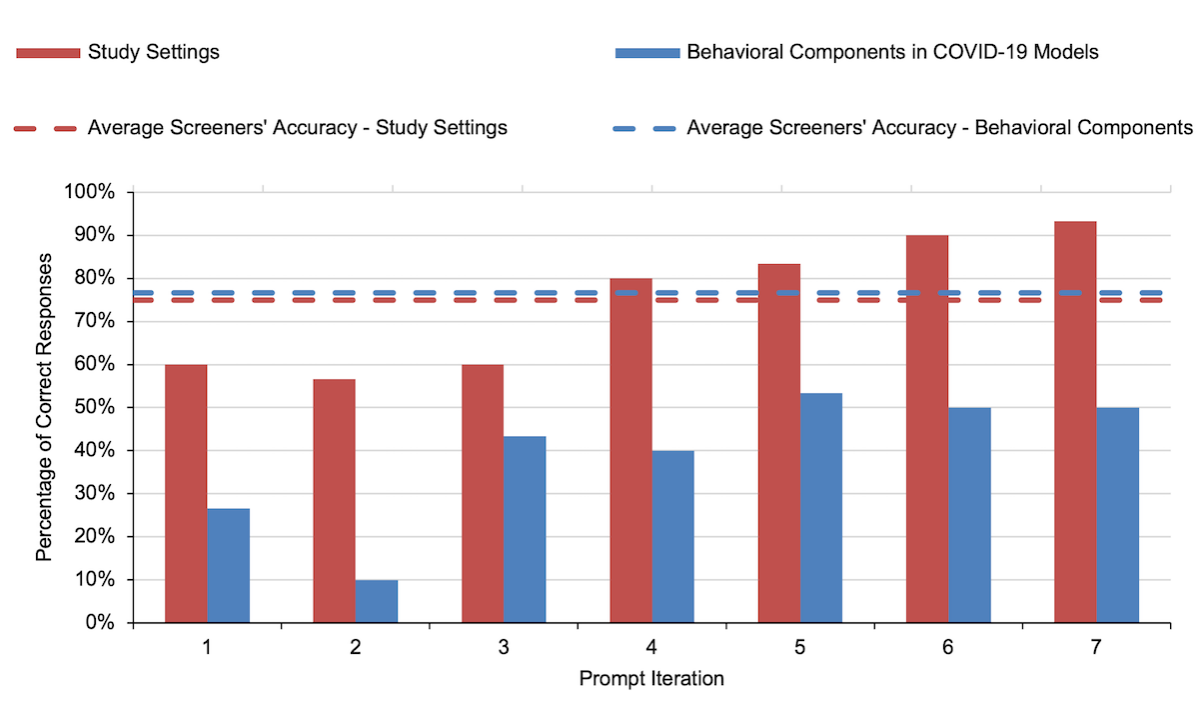
Average percentage of correct ChatGPT responses throughout iterations (bars) in comparison to the average accuracy of screeners before consensus (dashed lines) across study settings and behavioral components in COVID-19 models.

As a result of iterative prompt engineering, the average accuracy of ChatGPT’s responses showed a marked improvement of 33% and 23% between the initial and final iterations for extracting study settings and behavioral components, respectively (Figure 1). Specifically, in our initial prompts, 26 (43.3%) of 60 ChatGPT responses were correct. However, the latest prompt version yielded 43 (71.6%) correct answers. The iterative responses from ChatGPT and a comparison with our manual screening are detailed in Multimedia Appendix 1. By the fourth iteration, ChatGPT outperformed the individual screeners’ average accuracy in identifying study settings (Figure 1). However, ChatGPT consistently could not achieve a level of precision comparable to that of manual screeners (Figure 1) when extracting behavioral components.

**Table 2.** ChatGPT’s responses in the final version of the prompts.^a^

Study	Measure group 1: study settings			Measure group 2: behavioral components in COVID-19 models^b^		
	Location of analysis	Type of model, as presented by authors	Interventions analyzed	Changes in travel and mobility	Perception of risk and severity	Compliance and resistance to public health measures
Giordano et al [19]	Italy	Compartmental model known as the SIDARTHE model	Mass vaccination campaigns Nonpharmaceutical interventions Intermittent open-close strategies Different transmission rates due to new variants	A [B]^c^	B	B [A]
Tuomisto et al [20]	Helsinki University Hospital region in Finland	Agent-based model	Physical isolation Testing and tracing Mobility restrictions Health care capacity enhancement (Import of infections)^c^	D	B [A]	C [A]
Ashcroft et al [21]	Does not focus on a specific geographic region	Mathematical model	Quarantine Test-and-release strategies Reinforced hygiene adherence	A	A	C [D]
Sneppen et al [22]	Sweden	Agent-based model	Limiting social contacts Lockdown strategies Hygiene procedures	A	A	B [A]
Wong et al [23]	Hong Kong	Unspecified	Aggressive escalation of border control Implementing COVID-19 tests for overseas returners Quarantine measures and social distancing Active case finding	A [B]	A	B [A]
Gostic et al [24]	Unknown	Mathematical model	Symptom screening Risk screening	D [B]	A	A [D]
Kinoshita et al [25]	Unknown	Two-type branching process model	Contact tracing Case isolation	A	B	C [A]
Paul et al [26]	Emphasis on South Asia, including India, Bangladesh, and Pakistan	SEIR epidemic model	Lockdown Social distancing Individual-based precautionary measures	C [D]	D [A]	B
Ebigbo et al [27]	Unknown	Model-based on theoretical assumptions	Routine pre-endoscopy virus testing High-risk personal protective equipment use (Pre-endoscopy risk assessment questionnaire)	A	A	B [A]
Kim and Paul [28]	Unknown	Unspecified	Automated contact tracing Use of personal protective equipment Limited social distancing	A	A	C [D]

a All responses are shortened for presentation.

b Response categories: A, no mention; B, mentioned but not modeled; C, modeled exogenously but not analyzed; D, modeled exogenously and analyzed; E, modeled endogenously but not analyzed; F, modeled endogenously and analyzed.

c Brackets indicate manually screened responses, and parentheses flag ChatGPT’s additional incorrect info; both are used only in cells with incorrect responses.

**Figure 2.**
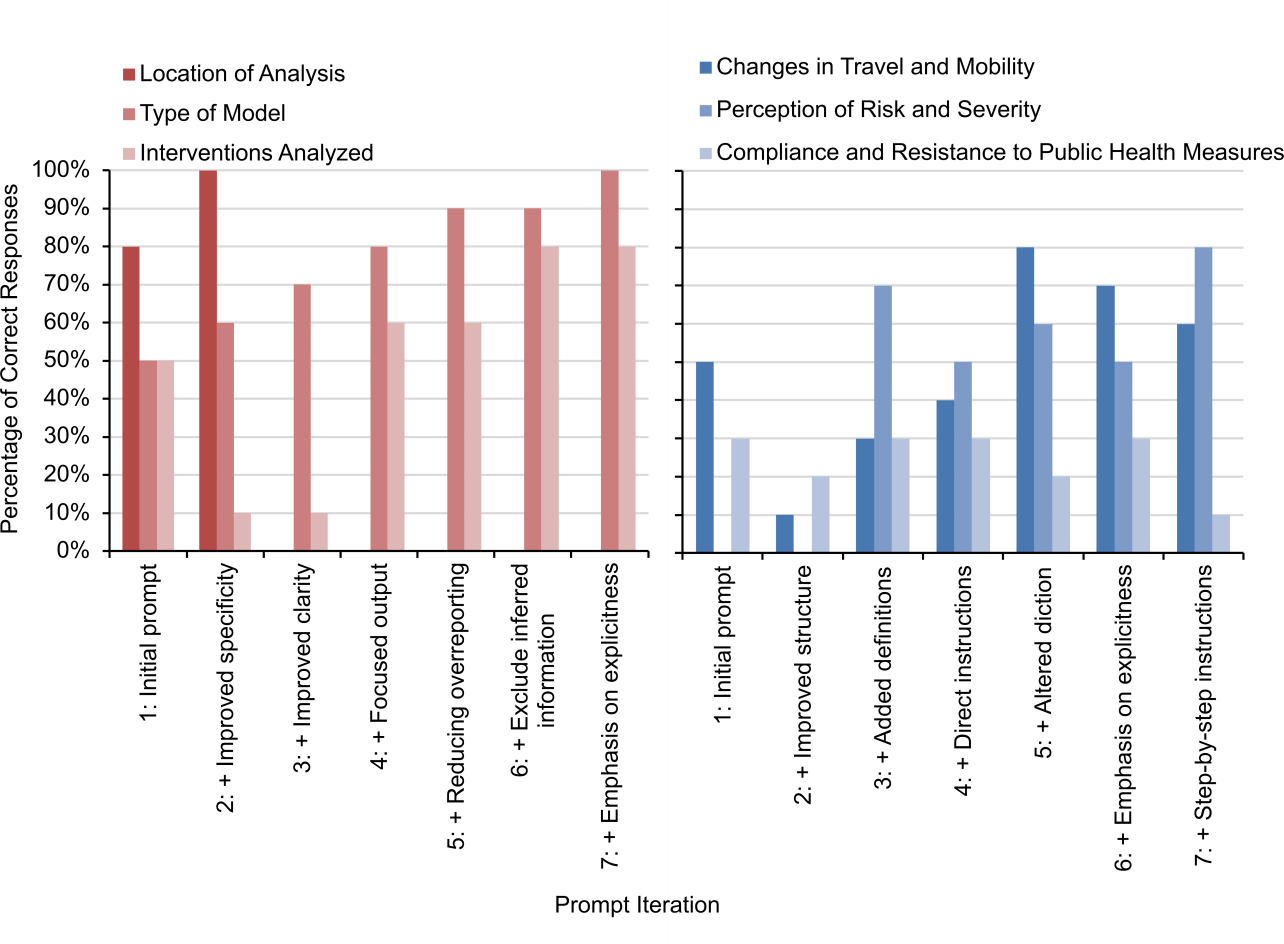
Percentage of correct ChatGPT responses throughout iterations of each prompt. The red bars depict the progression of ChatGPT’s accuracy for the three study setting elements, and the blue bars present the progression for the three behavioral component elements. Since the location of analysis achieved 100% by the second version, we concluded further iterations for this prompt.

Specifically, ChatGPT provided correct responses for all 10 papers [19-28] when prompted to identify the analyzed location and model type used by the second and seventh iterations of prompts, respectively. At most, ChatGPT correctly identified 8 of 10 interventions analyzed within the studies, achieving a peak accuracy of 80% by the sixth iteration (Figure 2). Conversely, it took 6 and 7 iterations to reach a peak accuracy of 80% (ie, 8 of 10 correct classifications) for classifying how each study assessed changes in travel and mobility and the perception of risk and severity, respectively. Our alignment with ChatGPT’s responses for coding compliance and resistance to public health measures only achieved a maximum consistency rate of 3 (30%) correct answers across the 10 studies [19-28] (Figure 2).

Finally, the contrast between the distribution of the manual coding and ChatGPT is presented in Figure S1 in Multimedia Appendix 1, and the distributions of ChatGPT’s responses are presented in Figure S2 in Multimedia Appendix 1.

## Discussion

### Principal Findings

Our analysis underscores the finding that ChatGPT’s assistance in full-text screening of study reports is particularly useful when handling simple inquiries, specifically general study settings, for which details are typically explicit in the text. However, the task of assessing nuances that necessitates drawing inferences (eg, the integration of human behavior in COVID-19 models), where the study may or may not have an explicit discussion, presents a significant challenge for ChatGPT.

Rather than prolonging the iterative process to elicit correct responses, we adopted a strategy of structuring prompts based on ChatGPT’s previous responses to ensure explicitness over the course of 7 iterations. Although it may have been possible to continue iterating to improve response accuracy, such a strategy is impractical in real-world scenarios. In addition, we acknowledge the potential risk of overfitting due to iterative prompt engineering on the same set of studies; however, our primary objective was to explore the utility of ChatGPT in real-world use cases where prompt refinement is common practice. This utility is especially relevant, given the broad reliance on SLRs to guide evidence-based practices across fields, with particular importance in health and biomedical sciences, where decisions impact health outcomes [31]. Hence, we limited this study to a manageable number of papers to better understand the limitations of prompt engineering itself. Importantly, the framework we used for evaluating ChatGPT (GPT-4) in COVID-19 modeling studies can be applied similarly to other SLRs regardless of the specific topic.

We highlighted that ChatGPT’s performance is influenced by the explicitness of information within the text, not just by the clarity or objectivity of the prompts. This underscores a nuanced limitation: the technology’s current dependency on explicit textual evidence for accurate data extraction. This limitation is notable, even with the use of straightforward prompts, underscoring a significant barrier in LLMs’ application to literature analysis. For instance, despite clear prompts, ChatGPT often struggled to correctly identify the model type in studies—expected to be straightforward—unless explicitly mentioned. Even when instructed to label model types as “unspecified” in the absence of clear documentation, early iterations often resulted in incorrect answers rather than adherence to the “unspecified” directive. This illustrates that ChatGPT’s accuracy is dependent not only on the prompt structure but also on the presence of explicitly detailed textual information. Hence, a central insight from this analysis is the significant obstacle that LLMs encounter when navigating ambiguity, further complicating the tasks of engineering effective prompts.

Furthermore, among ChatGPT’s responses that did not correspond to our manual coding, we observed a tendency for ChatGPT to extrapolate beyond the presented data. Despite instructions to confine responses to the explicit content of each study, ChatGPT often listed additional interventions not stated by the authors. This pattern of overreporting was also evident when categorizing studies based on the extent of their integration of behavioral components into the model. ChatGPT frequently assigned a higher integration level than that supported by the studies’ text (Multimedia Appendix 1). These skewed errors align with ChatGPT’s tendency to “hallucinate,” or provide confidently articulated yet factually unsupported responses [10].

Despite these challenges, LLM tools may be useful for SLRs. For 3 (30%) studies, ChatGPT correctly identified and analyzed interventions that we initially overlooked. For example, in Wong et al’s study [23], ChatGPT identified COVID-19 testing as an intervention in 6 of 7 prompt iterations—a detail our original manual assessment missed. This led us to reevaluate the study, and upon confirming ChatGPT’s accuracy, we modified our assessment accordingly. Conversely, we revisited our categorization of behavioral components in 6 incidences with which ChatGPT consistently disagreed, but we confirmed that our original manual coding was correct.

The main contribution of this study is to extend the understanding of current LLMs’ capabilities and limitations in handling complex data, providing valuable insights for those who conduct SLRs and are exploring the use of LLM platforms. Our observation that ChatGPT outperformed the average accuracy of individual reviewers when identifying study settings underscores its utility as an assistant or a second reviewer in extracting basic measures for SLRs. These results support previous research, which indicates the potential of LLMs for handling basic data extraction tasks effectively [10,11] and their use as a collaborator [32] or a second rater [33] in SLRs. However, for ChatGPT to be effectively used in this role, there remains a need for researchers to provide clear and detailed prompts that provide the relevant context. This approach requires researchers to have a thorough understanding of the context relevant to their inquiries and have access to reliable, coded data elements to directly compare against ChatGPT’s responses.

In terms of extracting complex components, ChatGPT failed to achieve comparable accuracy to that of individual screeners, highlighting the continued necessity of manual data extraction and additional research to overcome the limitations of this technology. Although further testing with a test sample may yield different results, the insufficient performance observed in our training data alone suggests that current LLMs remain unreliable for handling complex data extraction tasks. These findings align with other studies that discuss LLM performance, which similarly conclude that although automation is advancing, prevailing errors emphasize that structured oversight remains critical [15,34].

### Limitations

This study is subject to several limitations. First, this study focused on one aspect of the SLR process, given that other steps (eg, writing Boolean query formulations [35] and screening titles and abstracts [10,36]) have already been examined in greater detail. Second, we evaluated ChatGPT with only one review topic, which limits the generalizability of our results. Third, we often noticed inconsistencies in ChatGPT’s responses for the same prompts, but we did not formally assess reproducibility. Fourth, although accurate data extraction highly depends on the LLMs’ capabilities in accurately parsing PDF files [12], our paper does not quantify the impact of any errors in converting PDF files to text. Nonetheless, our results still highlight the stark differences in LLMs’ capabilities in accurately extracting simple versus complex data elements. Fifth, we selected ChatGPT for this study due to its wide accessibility and usability. We recognize that our exclusive focus is a limitation, as other models may have provided different insights or comparative performance benchmarks. Future research should explore the capabilities of multiple models to provide a more comprehensive understanding of LLM performance in this context. For example, certain advanced approaches (eg, retrieval-augmented generation) exist to better handle evidence or references from external sources. Since our focus was on a common, practical scenario in which typical reviewers use a publicly accessible GPT model, these specialized methods fall outside the scope of this study. Finally, due to the rapidly changing nature of LLMs, our findings may not hold over time.

### Conclusion

Overall, LLMs, and ChatGPT in particular, show promising performance in assisting the extraction of explicitly stated information from the full text of study reports, particularly when limited scientific reasoning is required. However, ChatGPT currently exhibits limited potential for fully automating data extraction across more complex, subjective measures. Our findings emphasize the ongoing necessity of human oversight in handling complex, nuanced data extraction tasks, even as LLMs continue to improve. This position is consistent with broader calls in the literature to adopt a cautious, well-evaluated approach to integrating LLMs into evidence synthesis workflows [15]. We highlight an important contribution to human-AI collaboration research, demonstrating the need to integrate AI tools with human oversight in SLRs, particularly in areas where current models fall short.

## Supplementary material

10.2196/68097 Multimedia Appendix 1 The iterative process of prompt engineering and additional results.

## Abbreviations

AI: artificial intelligence
API: application programming interface
LLM: large language model
SLR: systematic literature review

## References

[1] Owens JK (2021). Systematic reviews: brief overview of methods, limitations, and resources. Nurse Author Ed.

[2] Phillips V, Barker E (2021). Systematic reviews: structure, form and content. J Perioper Pract.

[3] Hossain MM (2024). Using ChatGPT and other forms of generative AI in systematic reviews: challenges and opportunities. J Med Imaging Radiat Sci.

[4] Mahuli SA, Rai A, Mahuli AV, Kumar A (2023). Application ChatGPT in conducting systematic reviews and meta-analyses. Br Dent J.

[5] Feng Y, Liang S, Zhang Y (2022). Automated medical literature screening using artificial intelligence: a systematic review and meta-analysis. J Am Med Inform Assoc.

[6] Blaizot A, Veettil SK, Saidoung P (2022). Using artificial intelligence methods for systematic review in health sciences: a systematic review. Res Synth Methods.

[7] de la Torre-López J, Ramírez A, Romero JR (2023). Artificial intelligence to automate the systematic review of scientific literature. Computing.

[8] Jonnalagadda SR, Goyal P, Huffman MD (2015). Automating data extraction in systematic reviews: a systematic review. Syst Rev.

[9] Fabiano N, Gupta A, Bhambra N (2024). How to optimize the systematic review process using AI tools. JCPP Adv.

[10] Khraisha Q, Put S, Kappenberg J, Warraitch A, Hadfield K (2024). Can large language models replace humans in systematic reviews? Evaluating GPT-4’s efficacy in screening and extracting data from peer-reviewed and grey literature in multiple languages. Res Synth Methods.

[11] Gartlehner G, Kahwati L, Hilscher R (2024). Data extraction for evidence synthesis using a large language model: a proof-of-concept study. Res Synth Methods.

[12] Konet A, Thomas I, Gartlehner G (2024). Performance of two large language models for data extraction in evidence synthesis. Res Synth Methods.

[13] Achter S, Borit M, Cottineau C, Meyer M, Polhill JG, Radchuk V (2024). How to conduct more systematic reviews of agent-based models and foster theory development - taking stock and looking ahead. Environ Model Softw.

[14] Li T, Higgins JP, Deeks JJ, Higgins JPT, Thomas J, Chandler J, Cumpston M, Li T, Page MJ, Welch VA (2023). Cochrane Handbook for Systematic Reviews of Interventions.

[15] Qureshi R, Shaughnessy D, Gill KAR, Robinson KA, Li T, Agai E (2023). Are ChatGPT and large language models “the answer” to bringing us closer to systematic review automation?. Syst Rev.

[16] Mostafapour M, Fortier JH, Pacheco K, Murray H, Garber G (2024). Evaluating literature reviews conducted by humans versus ChatGPT: comparative study. JMIR AI.

[17] Bijker R, Merkouris SS, Dowling NA, Rodda SN (2024). ChatGPT for automated qualitative research: content analysis. J Med Internet Res.

[18] Lee H, Mahmoudi H, Chang D, Jalali MS (2025). Review of human behavior integration in COVID-19 modeling studies. J Public Health (Oxf).

[19] Giordano G, Colaneri M, Di Filippo A (2021). Modeling vaccination rollouts, SARS-CoV-2 variants and the requirement for non-pharmaceutical interventions in Italy. Nat Med.

[20] Tuomisto JT, Yrjölä J, Kolehmainen M, Bonsdorff J, Pekkanen J, Tikkanen T (2020). An agent-based epidemic model REINA for COVID-19 to identify destructive policies. medRxiv.

[21] Ashcroft P, Lehtinen S, Angst DC, Low N, Bonhoeffer S (2021). Quantifying the impact of quarantine duration on COVID-19 transmission. Elife.

[22] Sneppen K, Taylor RJ, Simonsen L (2020). Impact of superspreaders on dissemination and mitigation of COVID-19. medRxiv.

[23] Wong MCS, Ng RWY, Chong KC (2020). Stringent containment measures without complete city lockdown to achieve low incidence and mortality across two waves of COVID-19 in Hong Kong. BMJ Glob Health.

[24] Gostic K, Gomez AC, Mummah RO, Kucharski AJ, Lloyd-Smith JO (2020). Estimated effectiveness of symptom and risk screening to prevent the spread of COVID-19. Elife.

[25] Kinoshita R, Anzai A, Jung SM (2020). Containment, contact tracing and asymptomatic transmission of novel coronavirus disease (COVID-19): a modelling study. J Clin Med.

[26] Paul A, Chatterjee S, Bairagi N (2020). Prediction on COVID-19 epidemic for different countries: focusing on South Asia under various precautionary measures. medRxiv.

[27] Ebigbo A, Römmele C, Bartenschlager C (2021). Cost-effectiveness analysis of SARS-CoV-2 infection prevention strategies including pre-endoscopic virus testing and use of high risk personal protective equipment. Endoscopy.

[28] Kim H, Paul A (2021). Automated contact tracing: a game of big numbers in the time of COVID-19. J R Soc Interface.

[29] Duarte F (2024). Number of ChatGPT users. Exploding Topics.

[30] Heston TF, Khun C (2023). Prompt engineering in medical education. International Medical Education.

[31] Schmidt L, Finnerty Mutlu AN, Elmore R, Olorisade BK, Thomas J, Higgins JPT (2023). Data extraction methods for systematic review (semi)automation: update of a living systematic review. F1000Res.

[32] Scherbakov D, Hubig N, Jansari V, Bakumenko A, Lenert LA (2024). The emergence of large language models (LLM) as a tool in literature reviews: an LLM automated systematic review. arXiv.

[33] Motzfeldt Jensen M, Brix Danielsen M, Riis J (2025). ChatGPT-4o can serve as the second rater for data extraction in systematic reviews. PLoS One.

[34] Chen X, Zhang X (2025). Large language models streamline automated systematic review: a preliminary study. arXiv.

[35] Wang S, Scells H, Koopman B, Zuccon G Can ChatGPT write a good Boolean query for systematic review literature search?.

[36] Syriani E, David I, Kumar G (2024). Screening articles for systematic reviews with ChatGPT. J Comput Lang.

