# Supplementary material for: Critical Assessment of Large Language Models’ (ChatGPT) Performance in Data Extraction for Systematic Reviews: Exploratory Study
Source: JMIR AI. 2025 Sep 11;4:e68097. doi: 10.2196/68097 (PMC12425462; doi:10.2196/68097)
Supplement: Multimedia Appendix 1 [file ai-v4-e68097-s001.pdf]

Multimedia Appendix 1

Iterative Prompt Engineering

An exhaustive list of our iterative process of prompt engineering for the six different measures is available in the accompanying Excel file, available at https://github.com/HesamVT/ChatGPT-Utility-Sys-Review. In addition, ChatGPT responses for each iteration of prompts in comparison to the correct answers can be found in the same file.

Overreporting in ChatGPT's Responses

Figure S1 marks the contrast between the distribution of the manual and ChatGPT's responses regarding classifying the behavioral components. In classifying 10 studies as no mention of behavioral components (A) across three behavioral measures, our manual coding identified 19 out of 30 evaluations as A (63%). In contrast, across seven iterations of the 10 studies, resulting in 210 prompts for extracting three behavioral measures, ChatGPT classified 54 as A, representing 26%. Contrarily, in classifying a study as modeling exogenously and analyzing a behavioral category (D), ChatGPT's responses significantly outnumbered those from manual coding. ChatGPT identified 78 out of 210 responses as category D (37%), compared to five out of 30 (17%) by manual coding.

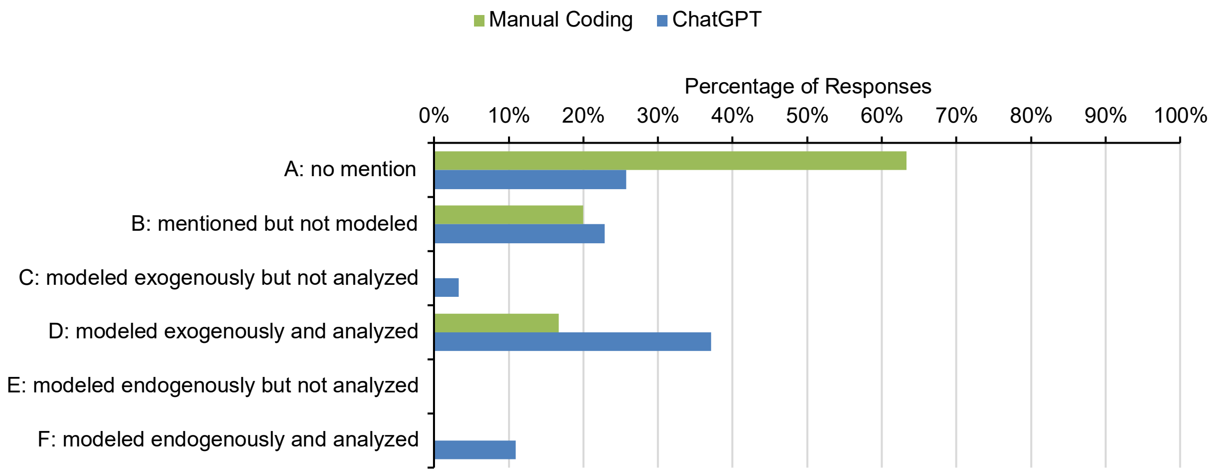

Figure S1. Comparison of distributions of coded human behavior classifications: manual coding vs. ChatGPT.

ChatGPT's tendency to overreport in its classifications, i.e., find mentions and inclusions in the model where they do not exist, is visible in **Figure S1**. To further highlight the overreporting issue, the distributions of ChatGPT's responses conditional on the manual coding being "A", "B", and "D" are presented in **Figure S2**. Among the 19 cases that do not even mention the specific human behavior (coded A), 67% of ChatGPT's responses point to a higher level of consideration of the human behavior in the paper (B to F). In contrast, among the five cases where the studies indeed include the specific human behavior in their model and analysis, only 28% of ChatGPT's responses underreport the level of consideration of the human behavior (A to C).

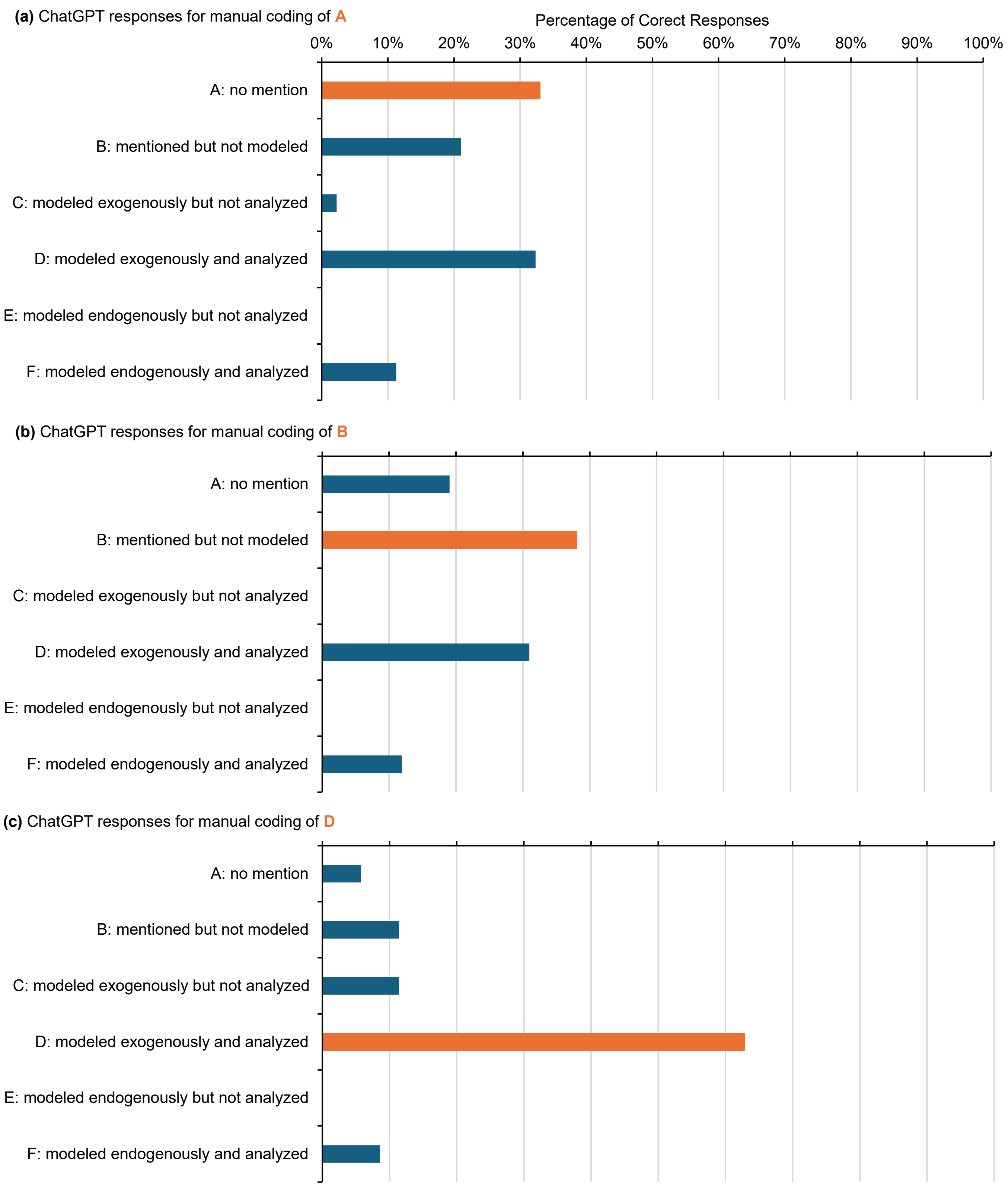

Figure S2. Distributions of ChatGPT's coded human behavior classifications conditional on the manual coding being (a) A: no mention, (b) B: mentioned but not modeled, and (c) D: modeled exogenously and analyzed.
